# Supplementary material for: Mycoparasitism capability and growth inhibition activity of Clonostachys rosea isolates against fungal pathogens of grapevine trunk diseases suggest potential for biocontrol
Source: PLoS One. 2022 Sep 6;17(9):e0273985. doi: 10.1371/journal.pone.0273985 (PMC9447919; doi:10.1371/journal.pone.0273985)
Supplement: Attachment — Submitted filename: Comments-PONE-D-22-11451.docx [file pone.0273985.s001.docx]

Comments−PONE-D-22-11451

This paper described a study of determining the effectiveness of Clonostachys rosea in controlling fungal pathogens causing grapevine trunk diseases (GTDs). The authors observed significant pathogen growth inhibition in both in vitro and in planta conditions. The reported findings contribute to the collective knowledge of biocontrol agents of C. rosea, especially with regard to in planta conditions. However, the following areas should be mentioned to improve the manuscript:

1. In abstract, experimental data should be provided to support the results and conclusion.
2. The authors identified eight pathogenic species of grapevine trunk diseases basing on the previous researches, therefore, the related references should be supplemented in the Table. Meanwhile, C. rosea strains were also isolated from the plants. We cannot regard them as beneficial microorganisms before a primary test of pathogenicity to grapevine is performed, especially some C. rosea isolates were noticed to significantly promote the growth of some GTD pathogens in vitro test (Table 2).
3. The authors should provide the context when presented the results. It is hard to fully understand the terms used in the results section, however, a short description of the study method and arrangement will help the readers to understand the data presented. Also, the results of Table 2 and Table 3 need to be clearly described in the text.
4. The authors thought the in planta and in vitro experiments suggest “in practice, antifungal compounds produced by C. rosea may be more important for effective biocontrol purposes than mycoparasitism”. It seems arbitrary, for the fungus was mainly found colonizing in soil and the base of the grapevine. In that case, induced resistance to the diseases is more involved, which has been verified in previous studies. It should be mentioned in discussion.
5. Standard three-line tables should be used in the manuscript with the SI units in the tables. The notes should be put below the table, separating with the title.
6. Supplement statistical analysis in the tables.
7. The figures should be improved. Fig. 1, which C. rosea isolate(s) was used? It should be marked. Fig. 2, clarify the arrows and change the bars to a line instead of an area. Fig.3, Rewrite the title to show the research content and put “× 10^6^” in the title of Y-axis. Different colors for the five isolates are not necessary.
8. Necrotic lesions were presented when inoculated with the pathogens (Fig.4), and the authors also mentioned that “while mock-inoculated plants did not show this symptom”. Here, an uninfected cutting should be presented together with the infected cuttings in the figure.
9. In discussion, the authors thought “only the C. rosea cells were stained by the MTT viability dye, suggests the necrotrophic nature of the parasitism”, and “lack of specific structures”. However, different infection structures have been observed in some mycoparasites, including C. rosea.
10. The authors should provide more information about the grapevine samples where the isolates were seperated, for example, the geographical location, cropping years, infected or not, status of soil.
11. In the section of methods, the experimental procedure should be described with more details, therefore, the readers can clearly follow your steps.
12. The term of “sporulation rate” is not correct.
13. The full names of genus should be used when first appear, then only abbreviations are required if not confused.

Furthermore, the authors should consider the following grammatical revisions:

L2-4. I would suggest to change the title to “Mycoparasitism capability and growth inhibition activity of Clonostachys rosea isolates against fungal pathogens of grapevine trunk diseases suggest potential for biocontrol”.

L14. Write the exact number of the pathogens instead of “several”.

L17-18. Change to “to the pathogens of Botryosphaeria dothidea and Diaporthe spp.”.

L19. Change “on in vitro grown colonies” to “on PDA plates”.

L21-22. “by inoculating… into the growth medium” is not consistent with the method.

L31-32. Can be revised to “chlorosis and necrosis occurrence on leaves, young shoots deformation, and necrotic spots appearance on the berries.”

L33-34. There are two mis-spelt words “widespred” and “occour”, and the sentence should be revised to “… the sudden death of the infected part or the whole plant may occur…”.

L47. Change “pest management” to “disease management”.

L81. In Table 1 use “strain number” instead of “strain ID”.

L86. It should be “Growth inhibitions of C. rosea strains …”.

L90. Change “percental radial growth inhibitions” to “growth inhibition rates”.

L115. Change to “Mycoparasitism of …”

L141. Cancel “were”.

L143. Change “developed by” to “caused by”.

L151. Change to “the development of vascular necrosis caused by…”.

L167. Change “This indicates…” to “This suggests…”.

L169-170. Change to “Our study is the first report…” .

L191. Cancel the word “therefore”.

L199. Change to “this fungus was also reported in the tissues of grapevine”.

L207. Cancel the word “species”.

L211. Change to “but susceptible to mycoparasitism”.

L218: Cancel the comma after the word “suggest”.

L251-252. Change “on cellophaned water agar (2 %m/v)” to “on 2% water agar covered with a piece of sterile cellophane”.

L266. Change to “dia. 3 mm”.
