# Supplementary material for: Mycoparasitism capability and growth inhibition activity of Clonostachys rosea isolates against fungal pathogens of grapevine trunk diseases suggest potential for biocontrol
Source: PLoS One. 2022 Sep 6;17(9):e0273985. doi: 10.1371/journal.pone.0273985 (PMC9447919; doi:10.1371/journal.pone.0273985)
Supplement: Attachment — Submitted filename: Response to Reviewers.docx [file pone.0273985.s002.docx]

Response to Reviewer#1’s comments

We would like to thank Reviewer#1’s thorough work, important comments and suggestions. We think that the manuscript has significantly been improved with the implementation of the recommended changes done. All changes in the manuscript are highlighted in red. Please find below our responses to Reviewer#1’s comments (in bold).

**Reviewer #1: Manuscript entitled "Mycoparasitic and growth inhibition capabilities of *Clonostachys rosea* isolates against grapevine trunk diseases fungal pathogens suggest potential for biocontrol" mainly talked about *C. rosea* isolates exhibited promising biocontrol ability against some grapevine trunk diseases. The manuscript is well written, and the results provide useful information for expanding the pathogen range by *C. rosea* biocontrol. Some points need to be addressed before it can be publication.**
**1. Line 63-69. Comprehensive review articles should be cited for introducing *Clonostachys rosea*. e.g. Sun et al. 2020 "Biology and application of *Clonostachys rosea*".**

The characterisation of *C. rosea* in the introduction was extended in the revised manuscript with a special focus on the biotechnological application of the fungus. Several additional papers were also cited.

**2. Line 75 and 232. Identification of fungal strains needs combined morphological characteristics and molecular sequencing. In line 232, the author declared that the identification of fungal strains would depend on morphological and molecular method. However, in Line 75, the results were mainly focus on ITS amplication. Then what about the morphological characteristics of these fungi? Especially the five C. rosea stains, are their morphological characteristics were same to known C. rosea strains?**

Yes, the morphological characteristics of the *C. rosea* isolates were in accordance with its previous description. Related text was added to the manuscript.

**3. Table 2. Why the significance analysis was only used for pathogens of *Eutypa lata*, *Phaeoacremonium minimum* and *Phaeomoniella chlamydospora*, but not for other pathogens in this Table?**

We used significance analysis to find out if a *C. rosea* strain is a more potent antagonist of a given pathogen than another *C. rosea* isolate. All data were analysed in this context, but in case of pathogens other than *E. lata, P. minimum* and *P. chlamydospora* there were no significant growth inhibition, or the *C. rosea* isolates did not differ significantly in the RGI% value.

**4. Line 98. Changed "antibiotic" to "antagonistic".**

The word is changed according to the suggestion.

**5. Line 105. Overgrow is a very important phenomenon for mycoparasitism. In this study, the author found the growth rate of Diaporthe strains were nearly not affect by *C. rosea*. Therefore, how about the overgrow behavior for *C. rosea* and *Diaporthe* strains? Just like in Fig.1 c and d, both two strains were overgrowth each other, or only *C. rosea* overgrowth pathogen strains? From Fig.1, it same like both the two strains are overgrowth each other.
Dose the single inoculation of *C. rosea* and pathogen strains were set as control in this study?**

Only the *C. rosea* strains grow over the *Diaporthe* spp. and *B. dothidea* colonies, the opposite can not been observed. Yes, single inoculated controls were also prepared, but have not been presented in the manuscript as figure. We thought that photographs of the dual cultures are appropriate to demonstrate the phenomena we wanted to show, and photographs of single inoculations would not provide additional information.

**6. Figure 1, why only (b) E. lata was investigated at 8 dpi, but other pathogens were at 15 dpi?**

The two different phenomena we wanted to demonstrate (antibiosis and overgrowth) were expressed at the highest level at different times in case of different pathogens. E.g. *P. chlamydospore* is very slow growing, therefore it needs more time for its mycelia to grow into the “effective antibiotic range” of *C. rosea* colonies. In case of *B. dothidea* and *Diaporthe* spp. antibiosis have not been detected and for the demonstration of overgrowth, older cultures were more suitable.

**7. Line 169-172. The sentence needs rewrote as "Our report is the first on the antagonistic activity of *C. rosea* against *E. lata*. Contrary to the findings of Silva-Valderrama..."
Confrontation test in plate could reflect the antagonistic activity of *C. rosea* against pathogens. However, antagonism had several mechanisms, and produce antibiotic is only one of the mechanisms. Therefore, from the confrontation test result, the author could provide conclusion of *C. rosea* had antagonistic activity against *E. lata*, but not antibiotic activity. Also the next sentence, *C. rosea* could produce many compounds with fungicidal activity, is not appropriate.**

Thank you for the suggestions, the text was rewritten according to your instructions.

Response to Reviewer#2’s comments

We are thankful for the overall positive opinion about our paper and the careful reading of our manuscript. We do believe that the manuscript has significantly been improved during the revision process and fulfills the requirements of publication. All changes in the manuscript are highlighted in red. Reviewer#2’s comments are in bold.

**Reviewer #2: This paper described a study of determining the effectiveness of *Clonostachys rosea* in controlling fungal pathogens causing grapevine trunk diseases (GTDs). The authors observed significant pathogen growth inhibition in both in vitro and in planta conditions. The reported findings contribute to the collective knowledge of biocontrol agents of *C. rosea*, especially with regard to in planta conditions. However, the following areas should be mentioned to improve the manuscript:**

**1. In abstract, experimental data should be provided to support the results and conclusion.**

A more detailed description of experimental conditions and results were included in the revised manuscript.

**2. The authors identified eight pathogenic species of grapevine trunk diseases basing on the previous researches, therefore, the related references should be supplemented in the Table. Meanwhile, *C. rosea* strains were also isolated from the plants. We cannot regard them as beneficial microorganisms before a primary test of pathogenicity to grapevine is performed, especially some *C. rosea* isolates were noticed to significantly promote the growth of some GTD pathogens in vitro test (Table 2).**

Suggested references were added to the table. Your concerns on the harmless of *C. rosea* to grapevines are understandable. However, we can assume the suitability of the application of this fungus on *V. vinifera* because of the following reasons:

1.)	The *C. rosea* isolates were obtained from healthy plants

2.)	There were no symptoms observed in our study on plants growing in media amended with *C. rosea*.

3.)	*C. rosea* is widely used on several crops without any undesired effects.

Point 1. and 2. were mentioned the revised manuscript.

**3. The authors should provide the context when presented the results. It is hard to fully understand the terms used in the results section, however, a short description of the study method and arrangement will help the readers to understand the data presented. Also, the results of Table 2 and Table 3 need to be clearly described in the text.**

A short description of the experimental setups were added to the results section in the revised manuscript, and table 2/3 are described with more details in the text.

**4. The authors thought the in planta and in vitro experiments suggest "in practice, antifungal compounds produced by *C. rosea* may be more important for effective biocontrol purposes than mycoparasitism". It seems arbitrary, for the fungus was mainly found colonizing in soil and the base of the grapevine. In that case, induced resistance to the diseases is more involved, which has been verified in previous studies. It should be mentioned in discussion.**

Thank you for the suggestion! The possibility of BCA induced resistance is mentioned in the discussion section with a reference.

**5. Standard three-line tables should be used in the manuscript with the SI units in the tables. The notes should be put below the table, separating with the title.**

Tables were reformatted according to your suggestions.

**6. Supplement statistical analysis in the tables.**

Where there were no significant differences, statistical groups were not labelled in the tables. We tried to reflect these cases in the text.

**7. The figures should be improved. Fig. 1, which *C. rosea* isolate(s) was used? It should be marked. Fig. 2, clarify the arrows and change the bars to a line instead of an area. Fig.3, Rewrite the title to show the research content and put "x10^6^" in the title of Y-axis. Different colors for the five isolates are not necessary.**

The suggested changes were done on the figures.

**8. Necrotic lesions were presented when inoculated with the pathogens (Fig.4), and the authors also mentioned that "while mock-inoculated plants did not show this symptom". Here, an uninfected cutting should be presented together with the infected cuttings in the figure.**

The suggested photographs were added to the figure.

**9. In discussion, the authors thought "only the *C. rosea* cells were stained by the MTT viability dye, suggests the necrotrophic nature of the parasitism", and "lack of specific structures". However, different infection structures have been observed in some mycoparasites, including *C. rosea*.**

Thank you for the suggestion, you are absolutely right. While we in fact did not observe specific infection structures, this does not mean that their lack indicates necrotrophic parasitism. The related sentence was removed from the manuscript.

**10. The authors should provide more information about the grapevine samples where the isolates were separated, for example, the geographical location, cropping years, infected or not, status of soil.**

The suggested information was added to the “Materials and methods” section.

**11. In the section of methods, the experimental procedure should be described with more details, therefore, the readers can clearly follow your steps.**

Additional details were added where it was possible.

**12. The term of "sporulation rate" is not correct.**

“Sporulation rate” was changed to “conidia production”.

**13. The full names of genus should be used when first appear, then only abbreviations are required if not confused.**

Corrections were done according to the suggestion.

**Furthermore, the authors should consider the following grammatical revisions:**

Thank you for the careful reading of our manuscript! All the grammatical corrections suggested below were done and the title was changed.

**L2-4. I would suggest to change the title to "****Mycoparasitism capability and growth inhibition activity of *Clonostachys rosea* isolates against fungal pathogens of grapevine trunk diseases suggest potential for biocontrol".**

**L14. Write the exact number of the pathogens instead of "several".**

**L17-18. Change to "to the pathogens of *Botryosphaeria dothidea* and *Diaporthe spp*.".**

**L19. Change "on in vitro grown colonies" to "on PDA plates".**

**L21-22. "by inoculating... into the growth medium" is not consistent with the method.**

**L31-32. Can be revised to "chlorosis and necrosis occurrence on leaves, young shoots deformation, and necrotic spots appearance on the berries."**

**L33-34. There are two mis-spelt words "widespred" and "occour", and the sentence should be revised to "... the sudden death of the infected part or the whole plant may occur...".**

**L47. Change "pest management" to "disease management".**

**L81. In Table 1 use "strain number" instead of "strain ID".**

**L86. It should be "Growth inhibitions of *C. rosea* strains ...".**

**L90. Change "percental radial growth inhibitions" to "growth inhibition rates".**

**L115. Change to "Mycoparasitism of ..."**

**L141. Cancel "were".**

**L143. Change "developed by" to "caused by".**

**L151. Change to "the development of vascular necrosis caused by...".**

**L167. Change "This indicates..." to "This suggests...".**

**L169-170. Change to "Our study is the first report..." .**

**L191. Cancel the word "therefore".**

**L199. Change to "this fungus was also reported in the tissues of grapevine".**

**L207. Cancel the word "species".**

**L211. Change to "but susceptible to mycoparasitism".**

**L218: Cancel the comma after the word "suggest".**

**L251-252. Change "on cellophaned water agar (2 %m/v)" to "on 2% water agar covered with a piece of sterile cellophane".**

**L266. Change to "dia. 3 mm".**
